# A Case of Refractory Hidradenitis Suppurativa Successfully Treated With Upadacitinib

**DOI:** 10.7759/cureus.85255

**Published:** 2025-06-02

**Authors:** Itsuki Takei, Satoko Shibata-Kikuchi, Ryoma Honda, Takeshi Tokito, Takeshi Nakahara

**Affiliations:** 1 Dermatology, Kyushu University, Fukuoka, JPN; 2 Dermatology, Kyushu Central Hospital, Fukuoka, JPN; 3 Dermatology, International University of Health and Welfare Narita Hospital, Chiba, JPN; 4 Orthopedic Surgery, Kyushu Central Hospital, Fukuoka, JPN

**Keywords:** adalimumab, hidradenitis suppurativa, psoriasis, secukinumab, upadacitinib

## Abstract

Hidradenitis suppurativa (HS) is a chronic, disabling inflammatory disease with a high unmet medical need, characterized by persistent, painful skin nodules, abscesses, and draining tunnels. Research on HS is rapidly evolving, with a variety of agents with different mechanisms of action being developed. As several Janus kinase/signal transducer and activator of transcription (JAK/STAT) signaling pathways are involved in the pathogenesis of HS, JAK inhibitors represent a promising therapeutic target. Herein, we present a case of severe hidradenitis suppurativa treated with upadacitinib following the failure of adalimumab and secukinumab.

## Introduction

Hidradenitis suppurativa (HS) is a chronic, disabling inflammatory disease with a high unmet medical need, characterized by persistent, painful skin nodules, abscesses, and draining tunnels. This disorder typically starts in the second or third decade of life. In Japan, the prevalence of HS, estimated from health insurance claims, is notably low at 0.0039%, contrasting with higher rates in Europe (1.0-4.1%) and the United States (0.1-0.2%). Japanese HS patients have different backgrounds from those in Western countries, and are characterized by male predominance, a higher incidence of Hurley stages II and III, higher PGA scores in patients with axillary lesions, and far fewer familial cases [1]. Research on HS is rapidly evolving, with a variety of agents with different mechanisms of action being developed, including adalimumab and bimekizumab, injectable monoclonal antibodies that inhibit tumor necrosis factor-alpha and IL-17, respectively. As several Janus kinase/signal transducer and activator of transcription (JAK/STAT) signaling pathways are involved in the pathogenesis of HS, JAK inhibitors represent a promising therapeutic target [2]. Herein, we present the first case of severe hidradenitis suppurativa treated with upadacitinib following the failure of both adalimumab and secukinumab.

## Case presentation

**Figure 1:**
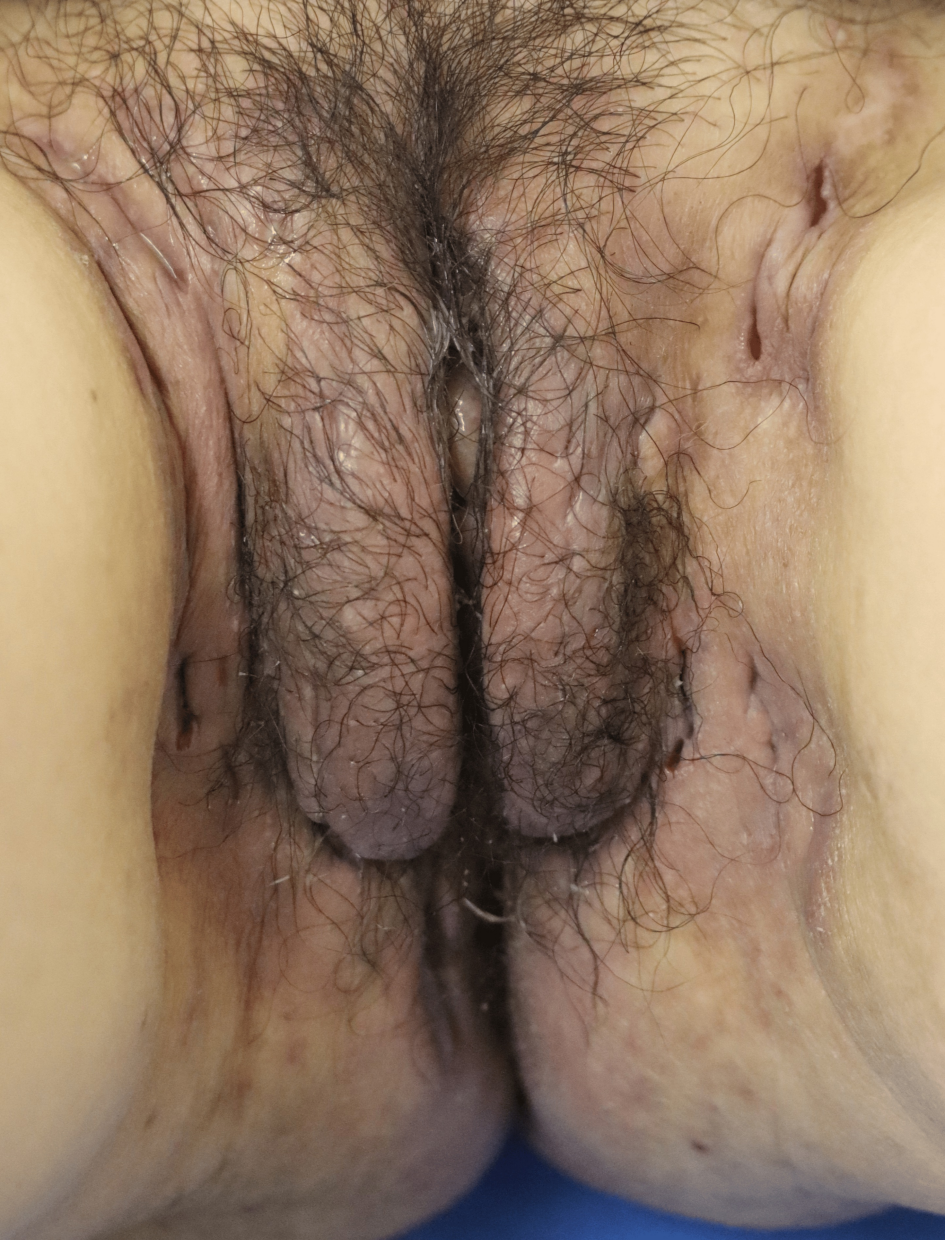
The abscesses and inflammatory nodules on the inguinal folds and intergluteal areas at the first visit.

**Figure 2:**
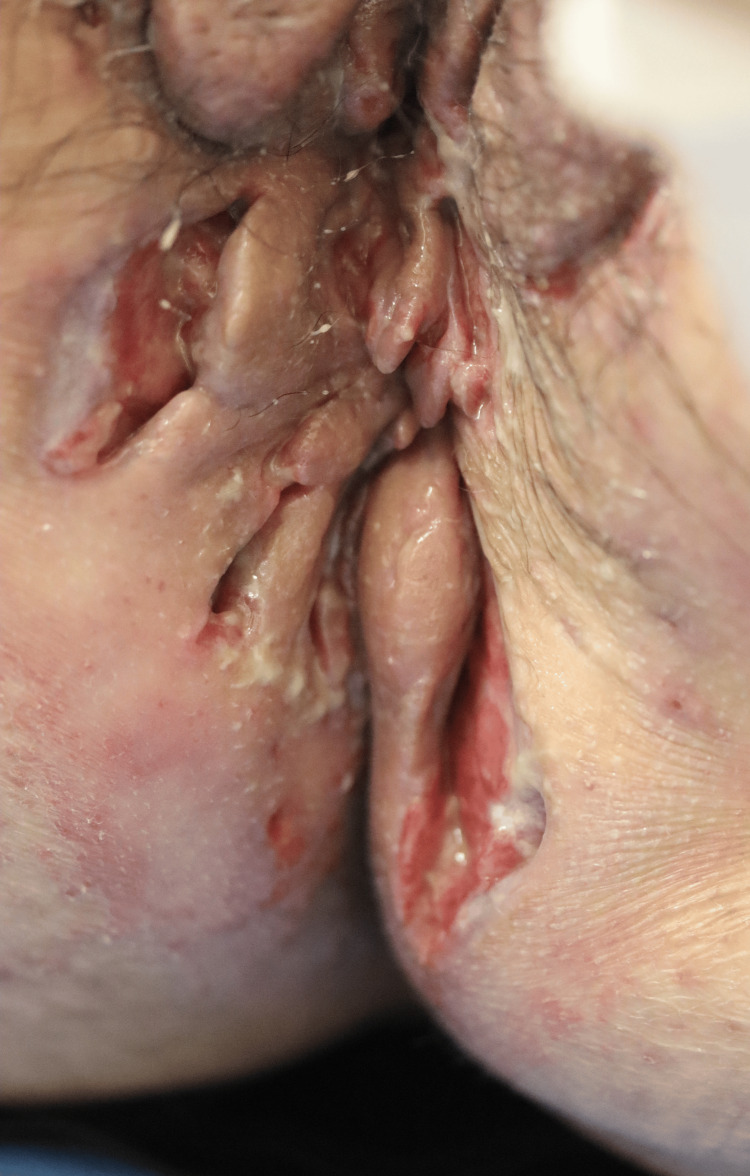
The abscesses and inflammatory nodules on the inguinal folds and intergluteal areas at the first visit.

A 50-year-old Japanese woman, who was a smoker and non-obese, visited our hospital for abscesses and inflammatory nodules on both axillae, inguinal folds, and intergluteal areas, with an overall International HS Severity Score System (IHS4) score of 36 (Figures 1, 2).

**Figure 3:**
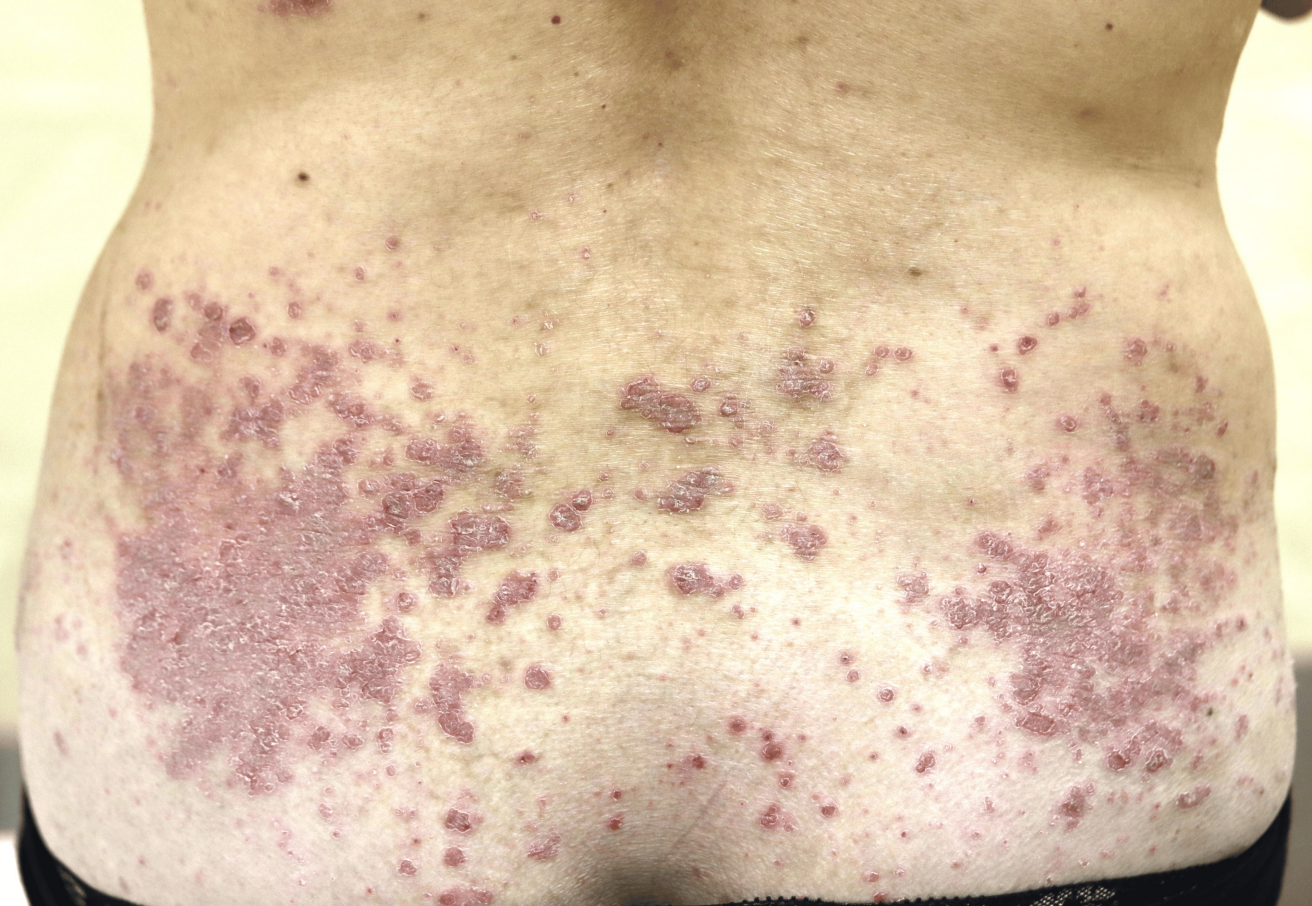
Erythema with scaling on the extremities and lower back appeared eight weeks after starting treatment with adalimumab.

Although the symptoms had been present for 40 years, she had never received treatment, and her condition had gradually worsened. In addition, she had developed right ankle pain seven months prior to her presentation. She visited an orthopedic rheumatologist, where rheumatoid arthritis was ruled out and celecoxib was started, but the ankle pain did not improve. One week after her first visit, adalimumab was initiated, and four weeks later, her IHS4 was 20 and the arthralgia had disappeared. A further four weeks later, however, erythema with scaling on the extremities and lower back appeared, and a skin biopsy was performed, which led to the diagnosis of psoriasis (Figure 3).

**Figure 4:**
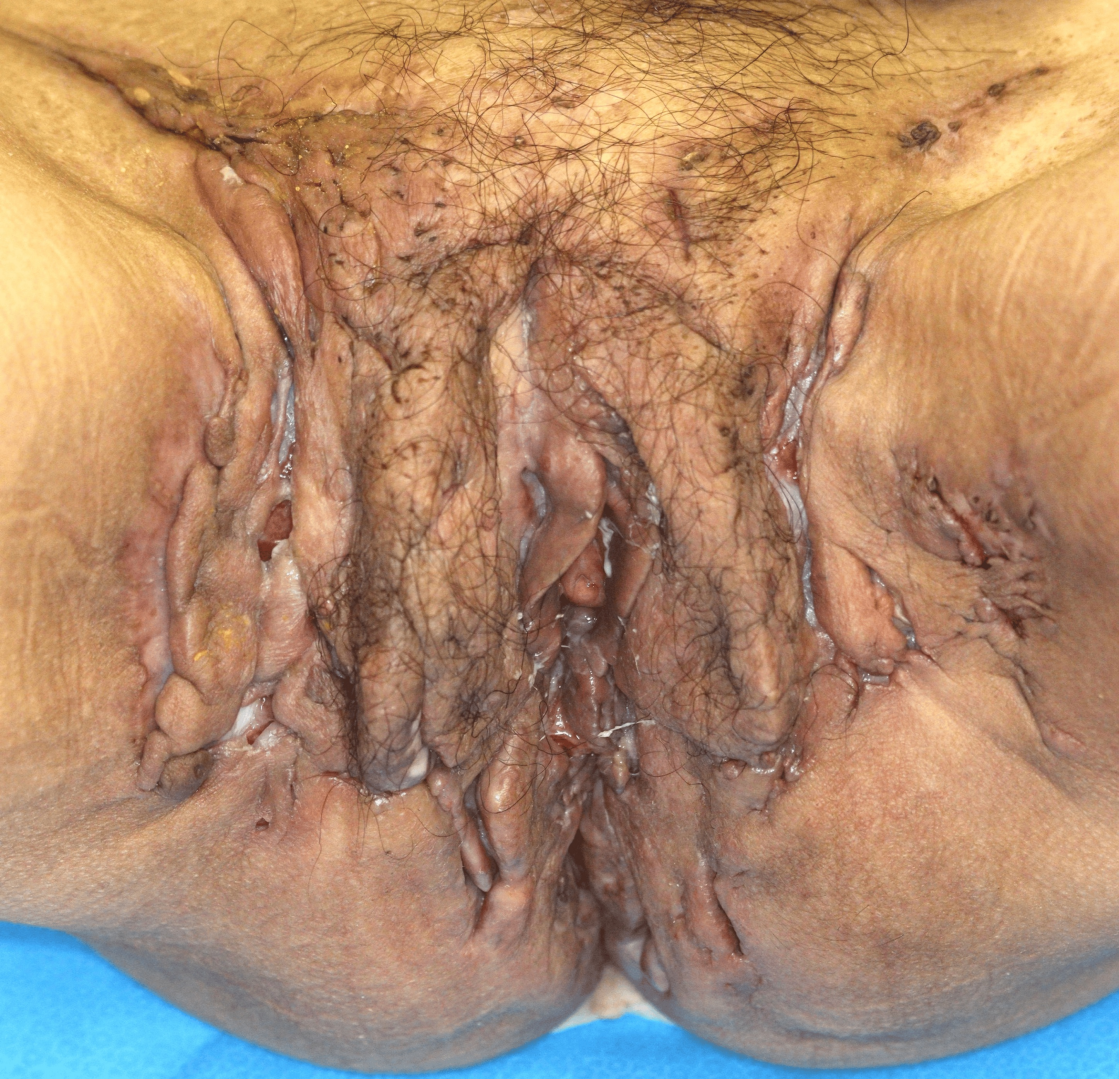
After self-discontinuation of adalimumab, switching to secukinumab resulted in improvement of psoriasis but exacerbation of HS symptoms, as well as the appearance of fever and diarrhea.

Calcipotriol hydrate betamethasone dipropionate resolved the rash, but the IHS4 score did not drop below 15. She refused to undergo surgery because she had to look after her children. Fifteen months after starting adalimumab, she self-interrupted it and developed right knee joint pain, while IHS4 rose to 24. Two months later, IHS4 was 28 and symptoms of psoriasis appeared on the bilateral lower legs, so secukinumab was started. After 16 weeks, the psoriasis resolved, but her IHS4 was 40, and secukinumab was discontinued due to worsening arthralgia, fever, and diarrhea (Figure 4). She had difficulty walking due to the arthralgia.

**Figure 5:**
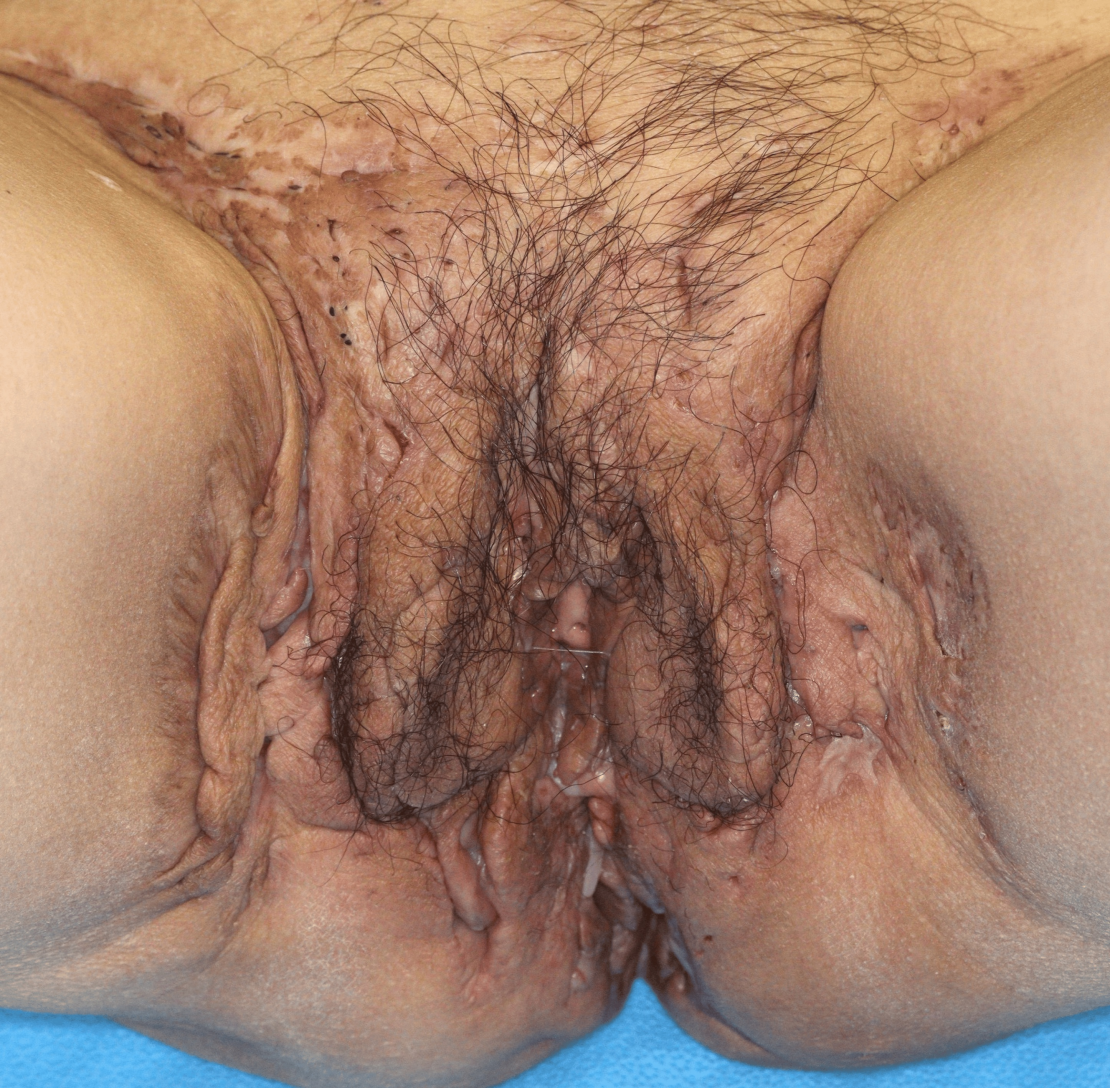
All symptoms improved after starting upadacitinib.

**Figure 6:**
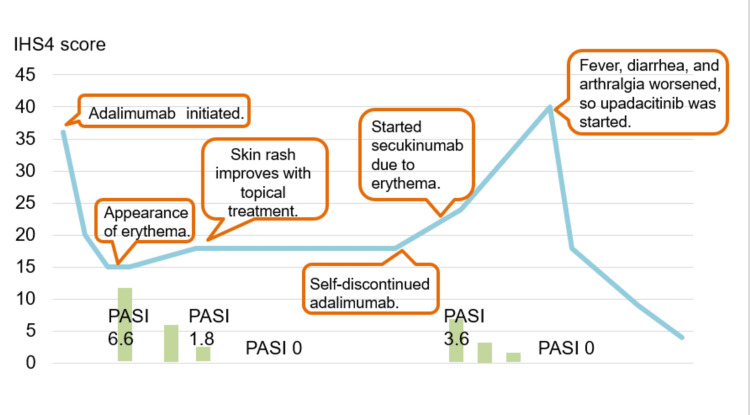
A graph showing the progress of the case's treatment and IHS4 score. IHS4:  International HS Severity Score System.

Following a consultation with the rheumatologist, a diagnosis of psoriatic arthritis was considered, and treatment with 15 mg of upadacitinib daily was initiated. By the following day, her fever had resolved, and both diarrhea and joint pain had improved. At the 28-week follow-up, she showed significant clinical improvement. Her IHS4 score was 4, and she reported no adverse effects (Figures 5, 6).

## Discussion

We hypothesized that our patient might have PsAPASH syndrome (psoriatic arthritis, hidradenitis suppurativa, acne, and pyoderma gangrenosum), a subtype of PAPA (pyogenic arthritis, pyoderma gangrenosum, and acne) syndrome, an autoinflammatory disease with three main features: pyogenic arthritis, pyoderma gangrenosum, and acne. In this case, there were complications of pyogenic arthritis and psoriatic arthritis, but no symptoms of pyoderma gangrenosum or acne. No mutation of the *PSTPIPI* gene, which is thought to cause PAPA syndrome, has been reported in PsAPASH syndrome, and it was not found in this case. Although the patient did not have all three of the primary features, it is unlikely that psoriatic arthritis and hidradenitis suppurativa occurred at the same time by chance, suggesting that some genetic abnormality or acquired condition may have caused a condition similar to PsAPASH syndrome.

Neither adalimumab nor secukinumab was effective in this patient with such complex symptoms, but upadacitinib was very effective. Although studies on the use of upadacitinib in HS are limited, phase 2 trials showed that 20 HS patients experienced an improved clinical response to 30 mg of upadacitinib [3]. Proteomic analysis connected upadacitinib to significant reductions in the expression of inflammatory pathways (such as IL-23, IL-10, and IL-12 signaling) associated with disease activity in HS [4, 5].

## Conclusions

In summary, we presented a case of severe hidradenitis suppurativa treated with upadacitinib following the failure of adalimumab and secukinumab. While upadacitinib is not currently approved for HS, this case report shows its potential for this complex inflammatory disease. With our case report, we hope to broaden the therapeutic options for HS and motivate further research to determine whether JAK inhibitors could play a role in HS treatment.
